# Editorial: Environmental stressors and metabolic disease

**DOI:** 10.3389/fendo.2023.1298687

**Published:** 2023-10-06

**Authors:** Kezhong Zhang, Cuiqing Liu, Marianna Sadagurski, Qinghua Sun

**Affiliations:** ^1^ Center for Molecular Medicine and Genetics, Wayne State University School of Medicine, Detroit, MI, United States; ^2^ Department of Biochemistry, Microbiology, and Immunology, Wayne State University School of Medicine, Detroit, MI, United States; ^3^ School of Public Health, Zhejiang Chinese Medical University, Hangzhou, China; ^4^ Department of Biological Sciences, Integrative Biosciences Center, Wayne State University, Detroit, MI, United States

**Keywords:** environmental stressor, metabolic disease, complex disease, air pollution, public health

Environmental challenges, such as air pollution, heavy metals, climate change, and overuse of natural resources, are closely associated with the development of modern human complex diseases (1). Epidemiological and animal model studies have linked environmental pollutants to the increase of mortality and morbidity associated with the progression of complex diseases. For the mechanistic understanding, disruption or dysregulation of immunity and/or metabolic homeostasis represent the major events that drive the pathogenesis of environmental complex diseases (2, 3). This has been consolidated by accumulating evidence provided by the biomedical and public health research communities in the past decades.

Understanding the effects and mechanisms by which environmental risk factors trigger or exacerbate pathogenesis has a high impact on the prevention and treatment of modern common human complex diseases. To this direction, we have assembled an issue of timely research and review articles with a focus on the adverse health effects and mechanistic basis of variable environmental stressors in diseases.

In this Research Topic, Xing et al. provided new light into the adverse health effect and the mechanism by which chronic constant light exposure exacerbates obesity-related kidney disease. With a rat model of high fat diet-induced renal injury, they demonstrated that constant light exposure exacerbated obesity, insulin resistance, dyslipidemia, as well as renal functional decline and fibrosis in high fat-fed rats by increasing renal hypoxia induced factor 1α (HIF1α) and decreasing in prolyl hydroxylase domain 1 (PHD1) and PHD2 expression in the kidney. In regard to the impact of benzene pollution in metabolic disease, Cui et al. investigated the effect of benzene exposure on adipose tissues using both *in vivo* and *in vitro* experiments. Coincident with dysregulated gene expression involved in lipogenesis and lipolysis, benzene exposed-male C57BL/6J mice exhibited reduced body fat content and adipokine levels due to lipoatrophy in white adipose tissue.

In terms of pathophysiological consequences of fine airborne particulate matter (PM_2.5_), an interesting research article by Zordao et al. described that maternal exposure to airborne PM_2.5_ impairs offspring’s energy metabolism and gut microbiota composition in a gender-dependent manner. The study concluded that exposure to PM_2.5_ during gestation is more harmful to metabolism than exposure during lactation. Male offspring had an unfavorable metabolic phenotype caused by PM_2.5_ exposure. In another work published in this Research Topic, Zhang et al. performed metabolomics and transcriptomics analyses with the liver tissues of PM_2.5_-exposed mice. Particularly, they demonstrated that PM_2.5_ exposure suppresses peroxisomal proliferative agent-activated receptor α (PPARα) and PPARγ but activates sterol regulator-binding protein 1 (SREBP1) in mouse livers. Their findings confirmed that airborne PM_2.5_ pollution represents a major environmental stressor that facilitates non-alcoholic fatty liver disease and type-2 diabetes (4–7). Additionally, a nationwide prospective cohort study in China performed by Du et al. confirmed the correlation between PM_2.5_ pollution and birth weight. Gestational PM_2.5_ exposure is closely associated with pre-pregnancy BMI and increased birthweight.

In this Research Topic, a review article by Koshko et al. highlighted the current understanding of the links between prenatal pollutant exposure and the hypothalamic programming of metabolism (8). The other review article by Roth and Petriello discussed the links between polyfluoroalkyl substances (PFAS) pollution and type 2 diabetes and the methods of statistical analyses to determine the links.

In summary, we anticipate that this collection of research and review articles from the front-running scientists will help the biomedical research community and public health professionals get a glimpse of new topics on the adverse health effects and mechanistic basis of environmental risk factors-caused illnesses. While much work in understanding the effects and mechanistic basis of environmental stressors in modern human complex diseases remains to be done, we hope that this Research Topic will trigger more interest in this important research direction.

## Author contributions

KZ: Conceptualization, Supervision, Writing – original draft, Writing – review & editing. CL: Validation, Writing – review & editing. MS: Validation, Writing – review & editing. QS: Validation, Writing – review & editing.
